# Hypertrophic olivary degeneration: a 7 Tesla advanced imaging case report

**DOI:** 10.3389/fnins.2025.1656655

**Published:** 2025-09-24

**Authors:** Tommaso Calzoni, Graziella Donatelli, Gianmichele Migaleddu, Marta Lancione, Paolo Cecchi, Laura Biagi, Michele Caniglia, Roberto Ceravolo, Mirco Cosottini

**Affiliations:** ^1^Department of Translational Research on New Technologies in Medicine, University of Pisa, Pisa, Italy; ^2^Imago 7 Research Foundation, Pisa, Italy; ^3^Neuroradiology Unit, Azienda Ospedaliero Universitaria Pisana, Pisa, Italy; ^4^IRCCS Fondazione Stella Maris, Pisa, Italy; ^5^Neurosurgery Unit, Azienda Ospedaliero Universitaria Pisana, Pisa, Italy

**Keywords:** 7 Tesla, MRI, hypertrophic olivary degeneration, Guillain Mollaret triangle, quantitative susceptibility mapping, diffusion tensor imaging, tractograhpy, ultra high field magnetic resonance

## Abstract

**Background and objectives:**

A 50-year-old patient developed ataxia, nystagmus, and palatal tremor. Conventional magnetic resonance imaging (MRI) revealed inferior olivary nuclei enlargement and hyperintensity in T2-weighted images, indicating hypertrophic olivary degeneration (HOD). The patient’s past medical history reported proton therapy for an VIII cranial nerve Schwannoma. Here, we aimed to investigate the potential alterations involving tracts and nuclei composing the dentato-rubro-olivary pathway (Guillain-Mollaret triangle) using an advanced ultra-high field (7 T) MRI protocol.

**Materials and methods:**

The patient underwent a 7 T-MRI brain exam, including a multi-echo gradient-echo sequence for quantitative susceptibility mapping and diffusion tensor imaging (DTI). The DTI dataset was elaborated for tractography and computation of tensor metrics.

**Results:**

7 T-MRI allowed the depiction of the brainstem tracts and nuclei composing the Guillain-Mollaret triangle. Both qualitative and quantitative analyses of these structures demonstrated damage to the right red nucleus and the dentato-rubral tracts bilaterally. These findings are consistent with the pathophysiology of HOD and were confirmed in a follow-up MRI.

**Discussion:**

This study highlights the capability of 7 T-MRI to depict and investigate brainstem substructures such as tracts and nuclei. To the best of our knowledge, this is the first study to depict all tracts composing the Guillain-Mollaret triangle and directly document their alterations in HOD.

## 1 Introduction

Hypertrophic olivary degeneration (HOD) is a form of multisynaptic-transneuronal degeneration of the inferior olivary nucleus (ION) secondary to damage of the afferent fibers to the ION of the Guillain-Mollaret triangle (GMT) (Lapresle, 1979; Wang et al., 2019). The GMT was described by Guillain and Mollaret in 1931. Its vertices are represented by the ipsilateral ION, the contralateral dentate nucleus (DN), and the ipsilateral red nucleus (RN). The sides of the GMT are represented by the olivo-dentate tract, the dentato-rubral tract (DRT), and the rubro-olivary tract (ROT), which connect the above-mentioned nuclei. Within the GMT, an afferent and an efferent pathway can be identified relative to the ION. The efferent pathway consists of fibers originating from the ipsilateral ION and reaching the contralateral DN through the medial portion of the contralateral inferior cerebellar peduncle (olivo-dentate tract). The afferent pathway fibers originate from the contralateral DN, travel through the contralateral superior cerebellar peduncle (sCP), and reach the ipsilateral RN, crossing the midline at the Wernekink commissure (DRT). At the level of the RN, a proportion of fibers synapse with neurons of the parvocellular portion of the RN, from which postsynaptic fibers originate to reach the ipsilateral ION via the ipsilateral central tegmental tract (CTT), as part of the disynaptic component of the dentato-olivary pathway. The remaining proportion of fibers does not synapse and turns downward to reach the ipsilateral ION through the ipsilateral CTT as the monosynaptic component of the dentato-olivary pathway. These descending fibers form the ROT.

HOD occurs secondarily to damage of the afferent component of the GMT and can be divided into unilateral and bilateral (Carr et al., 2015; Wang et al., 2019). Unilateral HOD is secondary to damage to the ipsilateral afferent component of the GMT, whereas bilateral HOD is determined by damage to the afferent component of both the GMTs. The main clinical features of HOD are ataxia, palatal tremor (1–3 Hz rhythmic contraction of soft palate), ocular myoclonus, nystagmus, and Holmes tremor (less than 4.5 Hz rest, action, and postural tremor) (Wang et al., 2019).

The diagnosis of HOD is clinical and radiological (Wang et al., 2019). Conventional brain magnetic resonance imaging (MRI) shows ION enlargement and T2-hyperintensity. In some cases, it can help identify the primary cause of damage to the afferent component of the GMT (Kitajima et al., 1994; Steidl et al., 2022; Wang et al., 2019).

## 2 Materials and methods

**Figure 1:**
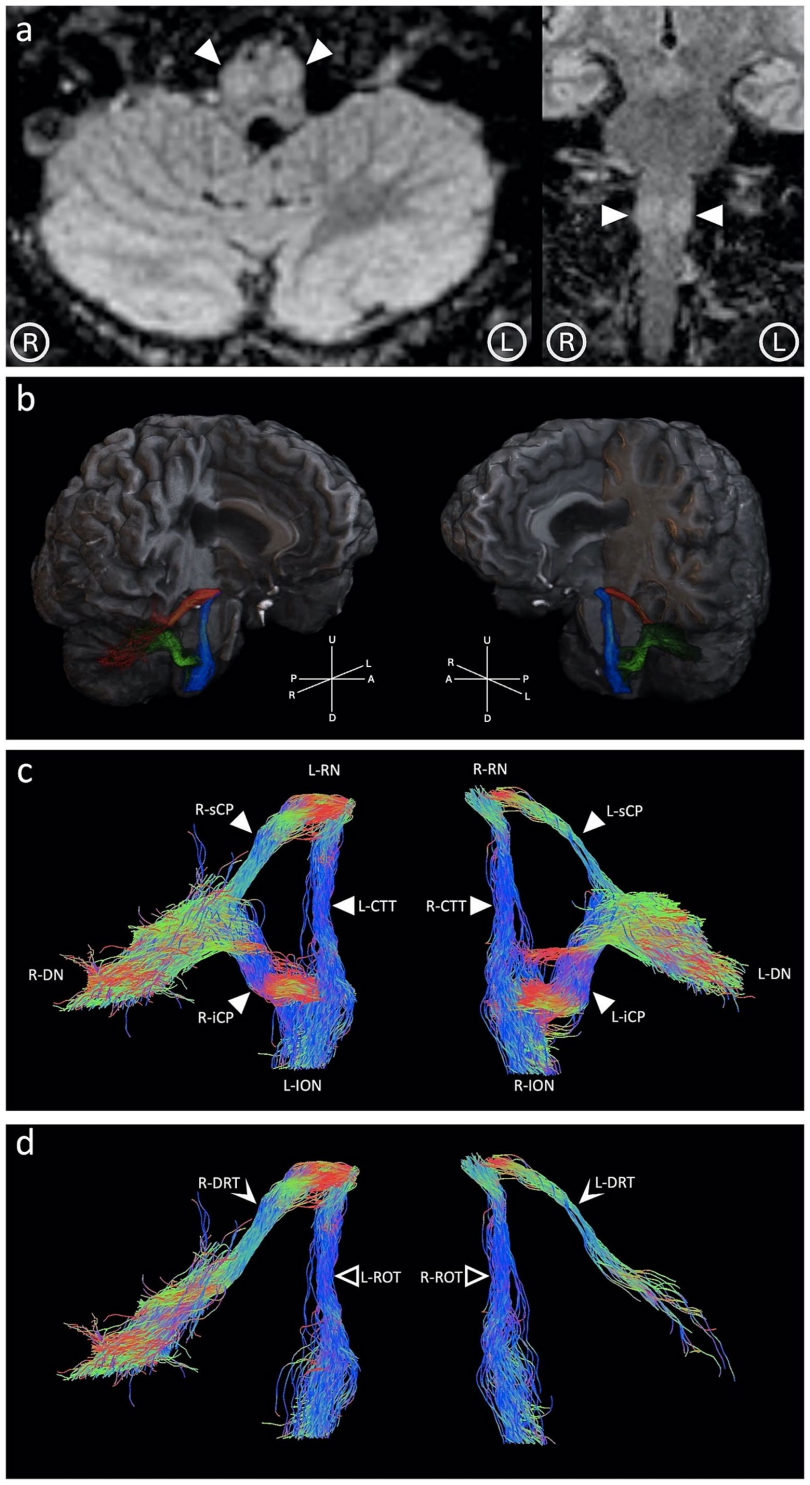
**(a)** Upon admission, 1.5 T MRI showing inferior olivary nuclei (arrow heads) enlargement and signal hyperintensity in axial and coronal T2-weighted Fluid Attenuated Inversion Recovery images. **(b)** The patient’s Guillain-Mollaret triangles, composed of the dentato-rubral tracts (depicted in red), the rubro-olivary tracts (depicted in blue), and the olivary-dentate tracts (depicted in green). **(c)** The corresponding directionally-color-coded (right–left: red; anterior–posterior: green; up-down: blue) tractography images showing the left dentato-rubral tract (L-DRT; 37 streamlines detected) and the right dentato-rubral tract (R-DRT; 1,360 streamlines detected) passing through the right and left superior cerebellar peduncles (R/L-sCP), the rubro-olivary tracts (ROTs) passing through the left and right central tegmental tracts (L/R-CTT), and the olivo-dentato tracts passing through the right and left inferior cerebellar peduncles (R/L-iCP). **(d)** The isolated afferent components to the inferior olivary nuclei (involved in the pathogenesis of hypertrophic olivary degeneration) are formed by the right and left dentato-rubral tracts (R/L-DRT) and rubro-olivary tracts (R/L-ROT).

A 50-year-old male patient was referred to our neuroradiology unit for progressive ataxia, bilateral nystagmus, and palatal tremor, which developed over 6 years, following proton therapy for a right VIII cranial nerve Schwannoma. Conventional 1.5 T MRI showed enlargement and signal hyperintensity in T2-weighted images of both the IONs (Figure 1a). These radiological findings, along with the suggestive clinical features, allowed the diagnosis of bilateral HOD (Wang et al., 2019).

At 43 years of age, an MRI was performed as part of the hydrocephalus follow-up and revealed a right intracanalicular VIII cranial nerve Schwannoma. The patient was referred to a proton therapy center where he underwent stereotactic proton therapy with a 50.4 GyRBE dose fractionated in 28 applications.

In the months following radiotherapy, the patient developed spontaneous bilateral vertical and horizontal nystagmus, associated with limitation in the adduction in both eyes, with nystagmus of the abducted eye and limitation in the upward gaze with retraction nystagmus. Hypometric and inaccurate saccades, along with smooth pursuit movement deficit, were present. Progressive ataxia, palatal tremor, dysarthria, and dysphagia for solids and liquids developed over 6 years following radiotherapy up to the moment of HOD diagnosis.

To directly investigate the tracts and nuclei of the GMT, a 7 T MRI was performed, given its promising results in the evaluation of the brainstem substructures (Donatelli et al., 2023).

One month after the 1.5 T MRI exam, the patient underwent an advanced 7 T MRI study protocol including a multi-echo gradient echo (GRE) sequence (voxel-size = 0.33 × 0.33 × 1.2 mm^3^) and diffusion tensor imaging (DTI) (voxel-size = 1.8 × 1.8 × 1.8 mm^3^; directions = 32; *b* = 0 and *b* = 1,000). From the phase of the GRE dataset, the quantitative susceptibility map was extracted using a Laplacian phase unwrapping algorithm, V-SHARP for background field removal, and iLSQR for dipole inversion implemented in STISuite (Li et al., 2014).

**Figure 2:**
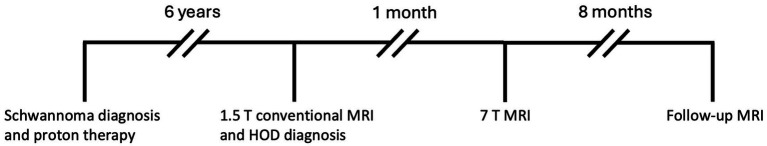
MRI examination timeline

The DTI dataset was elaborated using MRtrix3 (Tournier et al., 2019) to perform data preprocessing, including Eddy currents correction (Andersson et al., 2003), and to generate tractography images to extract fractional anisotropy (FA), radial diffusivity (RD), axial diffusivity (AD), and apparent diffusion coefficient (ADC) maps. ROIs were manually drawn in correspondence with the DNs, sCPs, and RNs. The iFOD2 probabilistic algorithm (Jenkinson et al., 2012) was used to generate streamlines connecting these structures (generating 5,000,000 random seeds per ROI). Exclusion ROIs were manually drawn in correspondence with the cerebral peduncles and immediately inferior to the IONs to exclude fibers comprising tracts not pertaining to the GMTs. For comparison, a healthy subject underwent the same 7 T-MRI study protocol, and the derived dataset was processed using the same techniques. The MRI examination timeline is reported in Figure 2.

## 3 Results

All tracts composing the GMT were successfully identified and isolated (Figures 1b,c). Tractography images showed a reduced representation of streamlines in the DRT compared to a healthy control. The DRT arising from the left DN (L-DRT) demonstrated a reduced number of streamlines compared to the contralateral one (R-DRT) (Figures 1c,d).

**Figure 3:**
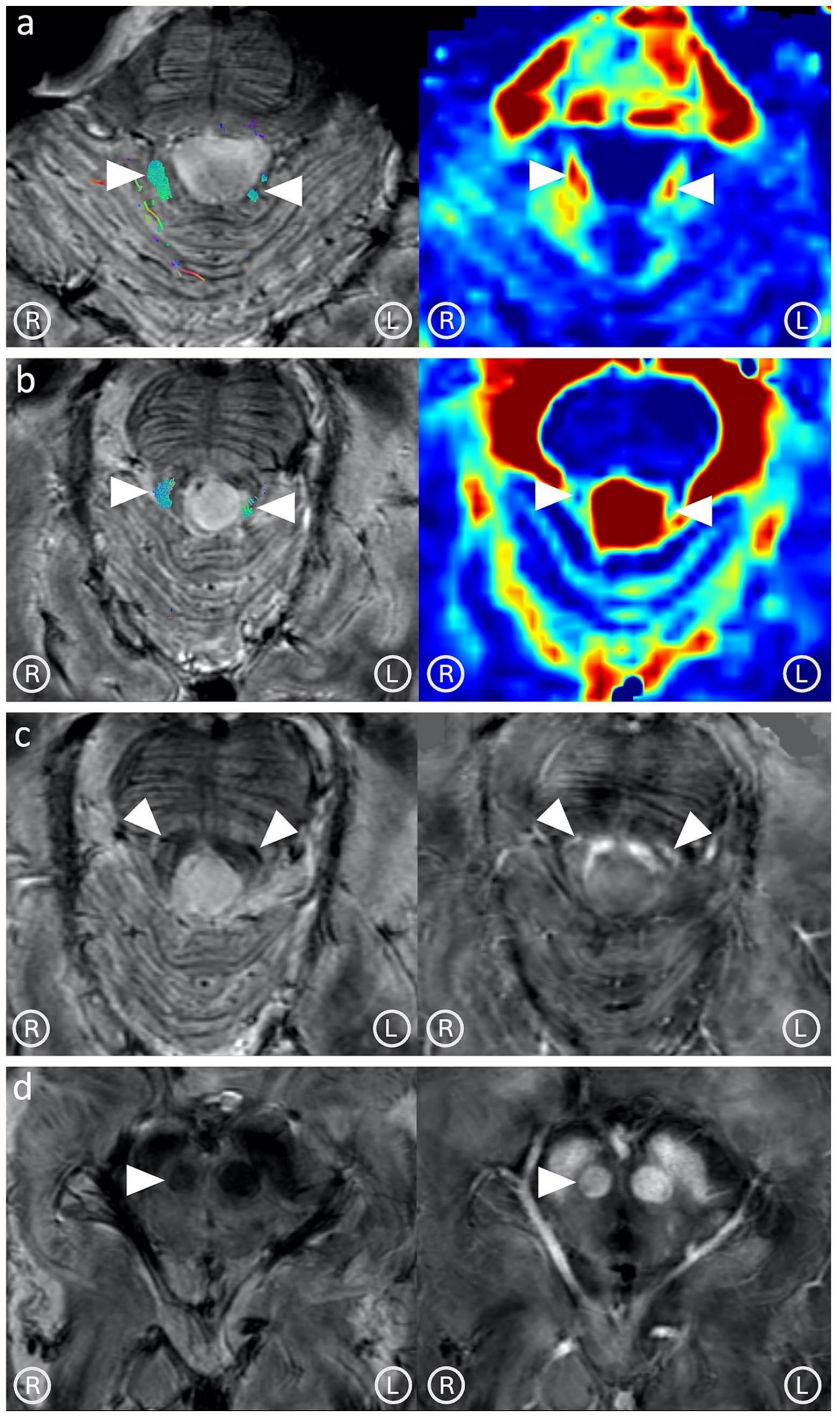
Tractography images and maps of fractional anisotropy (FA); **(a)** and radial diffusivity (RD); **(b)** show asymmetrical representation of the dentato-rubral tracts (arrow heads), with decreased FA and increased RD. Abnormally low signal in the T2*-weighted image and increased susceptibility in the corresponding quantitative susceptibility map **(c)** in correspondence with the fibers exiting the superior cerebellar peduncle (arrow heads), near the Wernekink commissure, suggesting the presence of iron-loaded activated microglia. The right red nucleus (arrowhead) is smaller than the contralateral **(d)**.

Along-tract DTI metrics were computed with respect to the FA, RD (mm^2^/s), and AD (mm^2^/s) maps. Both DRTs showed reduced FA (right:0.50; left:0.47) and increased RD (right: 0.00086; left: 0.00116) compared to FA (right: 0.63; left: 0.65) and RD (right: 0.00054; left: 0.00053) extracted from the control. The L-DRT had lower FA and greater RD than the R-DRT (Figures 3a,b), whereas AD and ADC were not significantly altered, as is common in Wallerian degeneration. The mean susceptibility value computed along each DRT tract was greater in the patient (right: 20.13 ppb; left: −4.39 ppb) than in the control (right: −5.97 ppb; left: −8.36 ppb). The unexpectedly low susceptibility value of the L-DRT can be explained by considering that these values are calculated along each streamline composing the tract; however, in the case of the L-DRT, the degeneration was such that very few streamlines were detected, which excluded areas with high susceptibility values from the computation (Figures 3a–c).

Both ROTs showed no significant alteration of FA, RD, AD, and ADC. No abnormalities were observed in the T2*-weighted image or the susceptibility map along their path through the CTTs.

The ROTs include the fibers composing the monosynaptic component of the dentato-olivary pathway; this theoretically implies that when DRTs are damaged, these fibers should also be affected. The absence of FA, RD, and AD abnormalities along both ROTs can be explained by considering that the monosynaptic fibers of the dentato-olivary pathway are dispersed in the CTTs and represent the minority of fibers at that level (Lapresle, 1979), making their changes not detectable.

**Figure 4:**
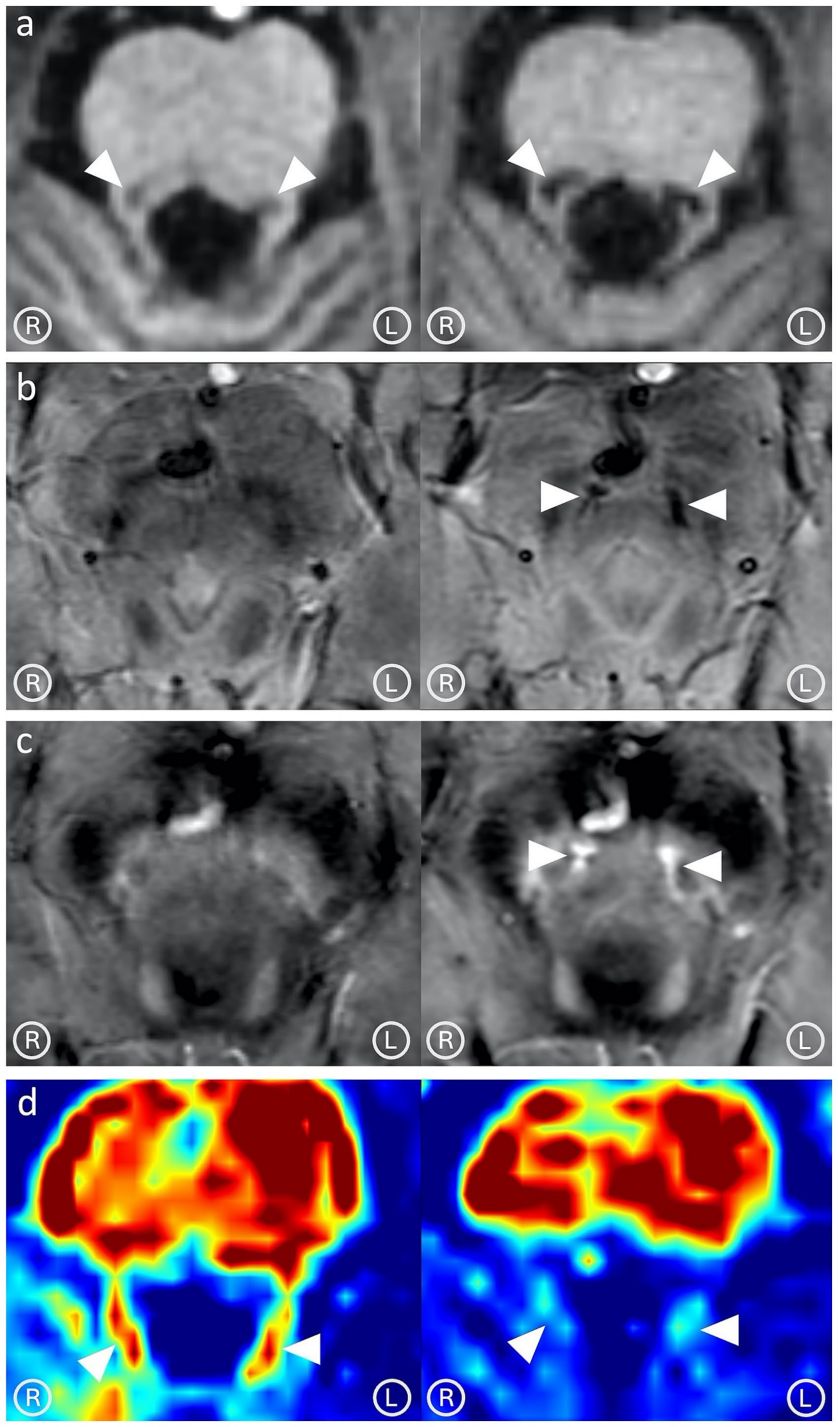
Comparison between the 7 T MRI (left column) and the follow-up MRI performed 8 months later (right column). Progression in the degeneration of the dentato-rubral tracts (DRTs) can be observed in T1-weighted images **(a)**. Abnormally low signal in the T2*-weighted image **(b)** and increased susceptibility in the corresponding quantitative susceptibility map **(c)** appeared further cranially along the DRTs’ course, near the Wernekink commissure. A significant decrease in fractional anisotropy was observed in correspondence with the DRTs **(d)**.

Abnormally low T2*-weighted signal and increased susceptibility were observed in the fibers exiting the sCP, suggesting the presence of activated iron-loaded microglia (Figure 3c). The right RN was smaller than the contralateral (Figure 3d). A follow-up MRI performed 8 months after the 7 T MRI study showed progression of the damage at the level of the DRTs (Figure 4), with a significant decrease in their FA values (right:0.26; left:0.25), evidence of gliotic-malacic sequelae at the level of DRTs, and increased susceptibility along the cranial portion of both DRTs.

## 4 Discussion

Radiological evidence of structural fiber damage (FA reduction and RD increase) and the suspicion of associated neuroinflammation (increased susceptibility in QSM map) indicated bilateral damage of the DRT (Cosottini et al., 2005), causing malfunction of the afferent component of the Guillain-Mollaret triangles and thus determining bilateral HOD (Wang et al., 2019). These findings were confirmed in the follow-up MRI.

Advanced 7 T MRI demonstrated the capability of depicting the tracts and nuclei composing the GMT.

Conventional MRI, on the other hand, showed no signal alteration along the DRT tracts, underscoring the superior sensitivity of 7 T MRI. Previous studies successfully documented DTI metrics alteration of the GMT in HOD patients by placing ROIs in correspondence with the presumed location of its tracts (Dinçer et al., 2011; Kitajima et al., 1994; Litkowski et al., 2012; Meoded et al., 2013; Orman et al., 2014; Shah et al., 2010). However, to the best of our knowledge, this is the first study in which all tracts composing the GMT were successfully depicted and investigated with a combination of advanced quantitative MR techniques.

Limitations to our study consist in it being a single case report and in the inherent error of DTI techniques for the reconstruction of tracts with multiple crossing fibers, such as the GMTs.

## Data Availability

The original contributions presented in the study are included in the article/supplementary material, further inquiries can be directed to the corresponding author.
